# Supplementary figures and images for: Galectin-3 Mediated Endocytosis of the Orphan G-Protein-Coupled Receptor GPRC5A
Source: Cells. 2025 Oct 9;14(19):1571. doi: 10.3390/cells14191571 (PMC12524283; doi:10.3390/cells14191571)

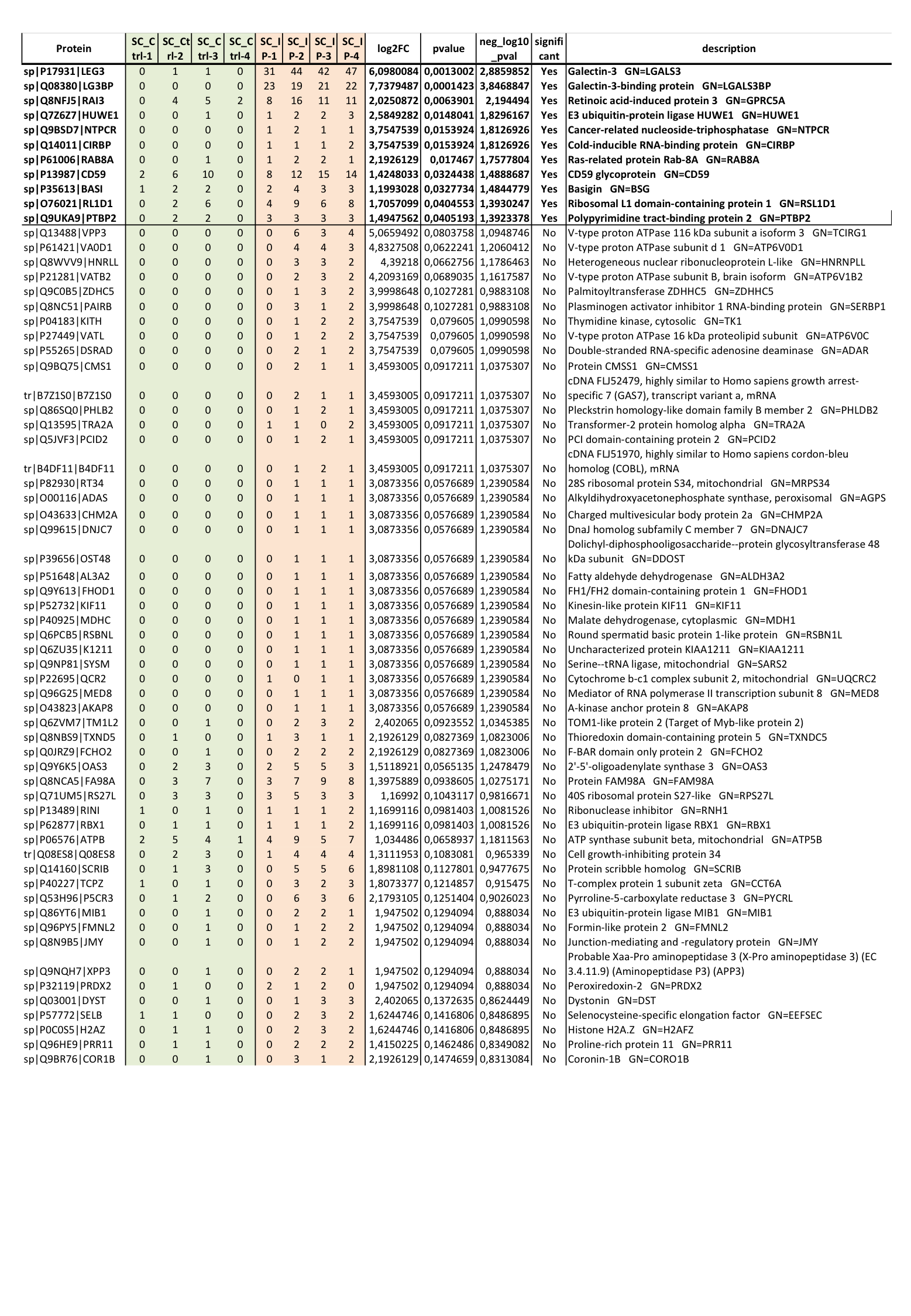

Supplement: Supplementary file 1 [file cells-14-01571-s001.zip › Figure S1 Boucheham Cells.tif]

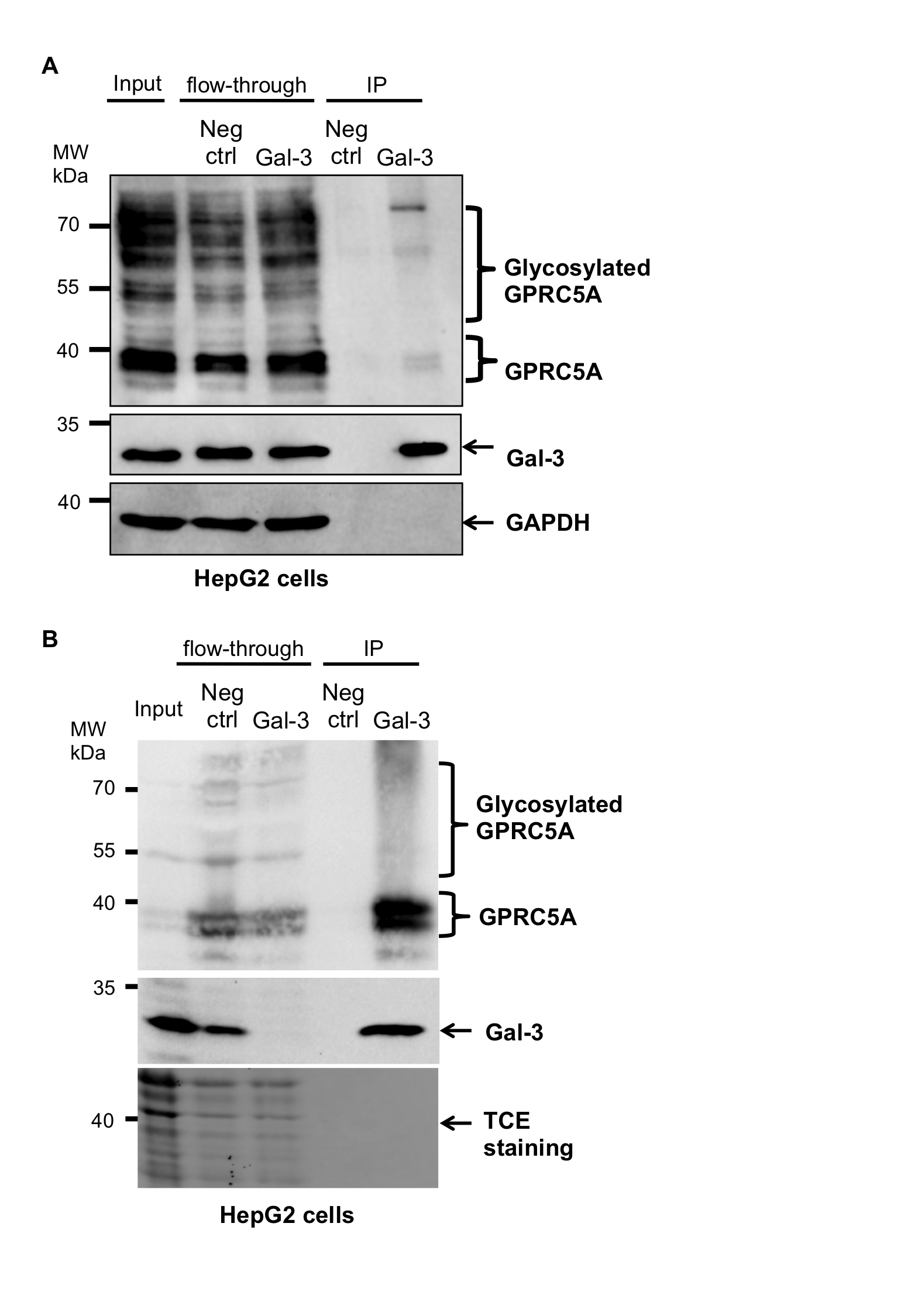

Supplement: Supplementary file 1 [file cells-14-01571-s001.zip › Figure S2 Boucheham Cells_revised WB coIP.tif]

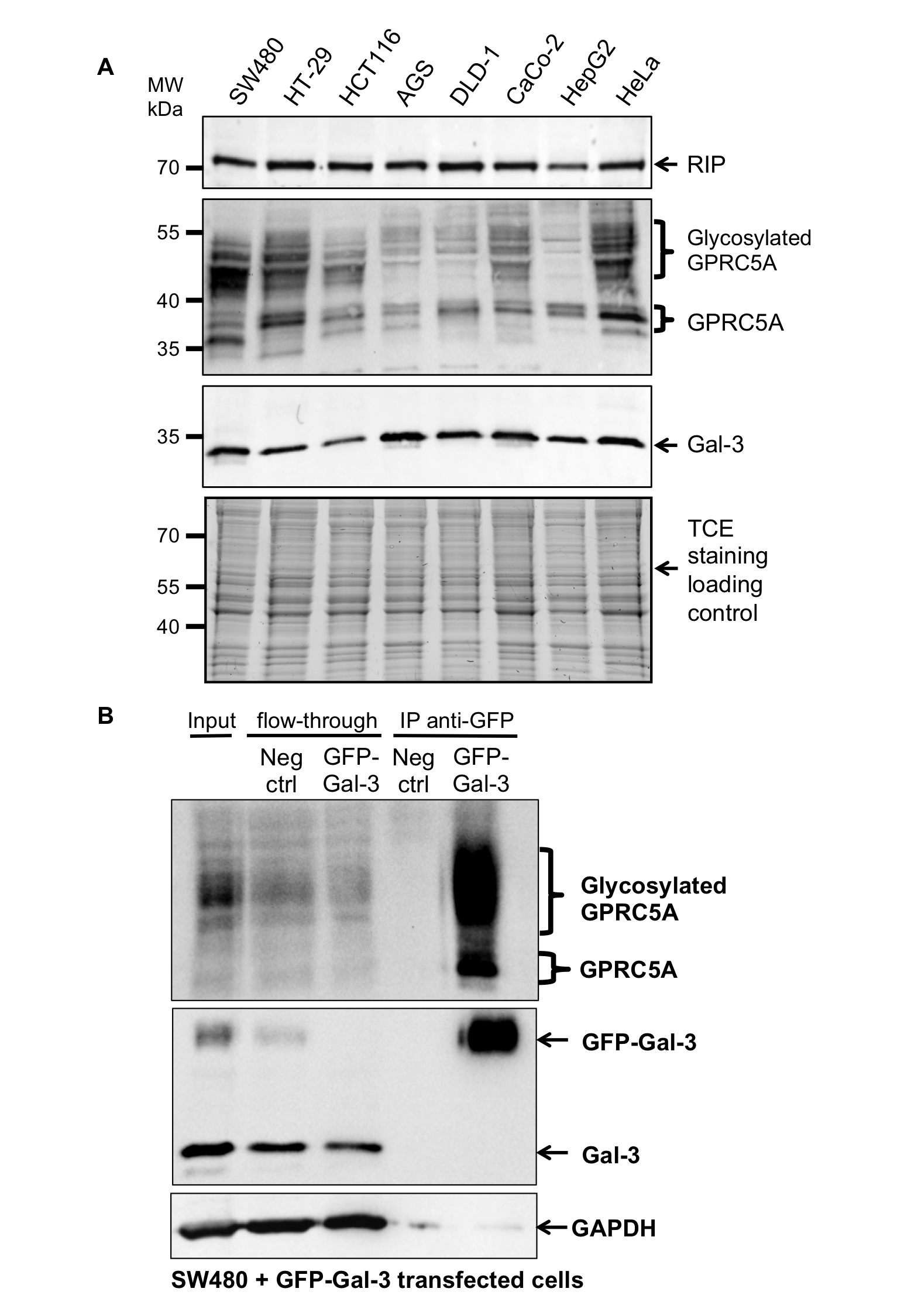

Supplement: Supplementary file 1 [file cells-14-01571-s001.zip › Figure S3 Boucheham Cells_revised.tif]

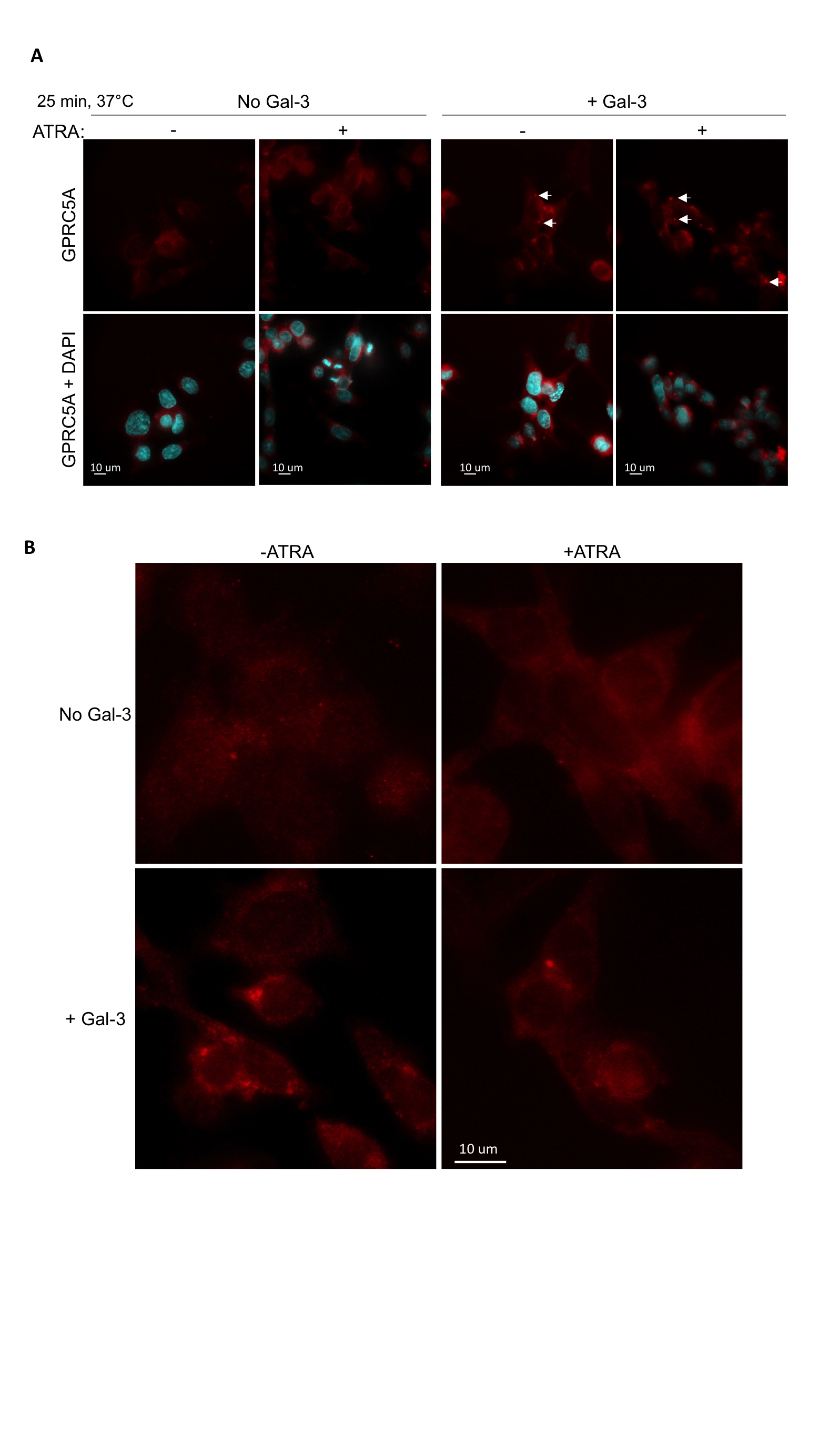

Supplement: Supplementary file 1 [file cells-14-01571-s001.zip › Figure S4 Boucheham Cells_revised.tif]
